# Propofol versus Dexmedetomidine for Sedation of Cancer Patients Undergoing Endoscopic Retrograde Cholangiopancreatography: Randomized Single-Blinded Controlled Study

**DOI:** 10.5812/aapm-148512

**Published:** 2024-09-09

**Authors:** Ahmed Mohamed Soliman, Yehya Mohamed Hamad, Abeer AbdElmonem Almaghraby, Ahmed Abdalla Mohamed, Shady Rady Abdallah

**Affiliations:** 1 Anaesthesia, ICU and Pain Management Department, National Cancer Institute, Cairo University, Giza, Egypt; 2 Anaesthesia, ICU and Pain Management Department, Faculty of Medicine, Cairo University, Giza, Egypt

**Keywords:** Propofol, Dexmedetomidine, Sedation, Cancer, Endoscopic Retrograde Cholangiopancreatography

## Abstract

**Background:**

Endoscopic retrograde cholangiopancreatography (ERCP) is a primary diagnostic and therapeutic option for pancreaticobiliary pathologies.

**Objectives:**

This study aimed to investigate the efficacy and safety of dexmedetomidine versus propofol during ERCP in cancer patients.

**Methods:**

This randomized controlled single-blinded trial was conducted with 202 cancer patients aged 21 to 60 years, of both sexes, with a body mass index of 18.5 to 30 kg/m^2^, and classified as American Society of Anesthesiologists (ASA) class II - III, who were undergoing ERCP. The patients were randomly assigned to two equal groups. The Propofol Group (n = 101) received a loading dose of propofol (1 - 2 mg/kg over 30 seconds) followed by an infusion (0.05 - 0.1 mg/kg/hour). The Dexmedetomidine Group (n = 101) received a loading dose of dexmedetomidine (1 μg/kg over 10 minutes) followed by an infusion (0.2 - 0.7 μg/kg/hour). The maintenance dose was adjusted during the procedure based on vital signs, Bispectral Index (BIS), and oxygen saturation.

**Results:**

The Dexmedetomidine group showed a significantly lower incidence of intra-procedural hypoxemic events (14.9% vs. 26.7%, P = 0.037) and a comparable incidence of hypotension (17.8% vs. 13.9%, P = 0.441). Dexmedetomidine also demonstrated significantly lower intraoperative pain scores according to the Facial Pain Score (P < 0.05), significantly lower postoperative Visual Analog Scale (VAS) scores (P < 0.05), and a lower frequency of postoperative nausea and vomiting (PONV) compared to the Propofol group. Additionally, there was a significantly higher frequency of endoscopist satisfaction in the Dexmedetomidine group compared to the Propofol group (P < 0.05).

**Conclusions:**

Dexmedetomidine can be used as a safe and effective alternative to propofol for deep sedation of cancer patients undergoing ERCP. It is associated with a lower incidence of hypoxemic events, effective intraoperative sedation, quicker recovery, and superior analgesic effects both intraoperatively and postoperatively compared to propofol.

## 1. Background

Endoscopic retrograde cholangiopancreatography (ERCP) is now a primary diagnostic and therapeutic option for pancreaticobiliary pathologies (1). Performed in the semi-prone position, the procedure typically lasts between 30 and 60 minutes (2). Cancer patients undergoing ERCP are often in poor overall health and may struggle with pain, discomfort from the position, anxiety, and nausea if not adequately sedated (3). Consequently, general anesthesia or moderate to profound sedation is frequently employed during ERCP procedures (4). However, general anesthesia often results in longer preparation times, anesthetic induction, tracheal intubation, and recovery, leading to increased procedural duration and cost (5). Some facilities use deep sedation as an alternative to general anesthesia, which, under an anesthesiologist’s supervision, can offer improved operating conditions and save time compared to general anesthesia (6, 7).

Propofol is commonly used in day-case procedures due to its rapid onset and short half-life, allowing patients to resume normal mental activities shortly after intravenous administration. However, higher doses of propofol can lead to adverse effects such as hypotension and hypoxia, which are common during upper gastrointestinal (GI) endoscopy. Prolonged hypoxia is a significant concern as it can lead to cardiac arrhythmia and coronary ischemia. Additionally, propofol's analgesic effects are insufficient to manage visceral traction pain (8).

Dexmedetomidine, a selective alpha-2 receptor agonist with sedative and analgesic properties, is increasingly replacing propofol in conscious sedation (9). A recent meta-analysis indicates that dexmedetomidine provides superior sedation for gastrointestinal endoscopy compared to traditional sedatives without increasing the risk of cardiac or respiratory complications (10).

To the authors' knowledge, there are few studies comparing dexmedetomidine and propofol in cancer patients undergoing ERCP. 

## 2. Objectives

This research aims to investigate the efficacy and safety of these two agents during ERCP in cancer patients, focusing on hemodynamic, respiratory, sedative, and cognitive functions.

## 3. Methods

This randomized controlled single-blinded trial involved 202 patients with malignant biliary obstruction undergoing ERCP for biopsy and cytology. The patients were recruited from the surgical oncology departments of the National Cancer Institute and the Faculty of Medicine at Cairo University. They were aged 21 to 60 years, with a body mass index of 18.5 to 30 kg/m², and classified as American Society of Anesthesiologists (ASA) class II - III. The study was approved by the Ethical Committee of Cairo University's Faculty of Medicine (Approval code MS-211-2023). All patients provided signed informed consent.

Exclusion criteria included sensitivity to the medications used in the study, use of any anticoagulants, neurological disorders, advanced liver or kidney disease, and psychiatric disorders.

Using computer-generated random numbers contained in opaque closed envelopes, the patients were randomly divided into two equal groups by an independent statistician. The Propofol Group (n = 101) received a loading dose of propofol at 1 - 2 mg/kg over 30 seconds, followed by an infusion of 0.05 - 0.1 mg/kg/hour. The Dexmedetomidine Group (n = 101) received a loading dose of dexmedetomidine at 1 μg/kg over ten minutes, followed by an infusion of 0.2 - 0.7 μg/kg/hour. The maintenance dose was adjusted during the procedure based on vital signs, BIS, and oxygen saturation. 

### 3.1. Intra-procedural

Throughout the procedure, all patients were continuously monitored using electrocardiogram (ECG), non-invasive blood pressure, and peripheral arterial oxygen saturation. Oxygen was administered via a nasal mask at a rate of 6 L/min. The Bispectral Index (BIS) was used to assess the level of sedation every five minutes. A BIS score greater than 90 indicated the patient was awake; a score between 71 and 90 indicated mild to moderate sedation; a score between 61 and 70 indicated deep sedation; and a score between 40 and 60 indicated general anesthesia. The target sedation level was a BIS score of 61 - 70.

The patient's vital signs were recorded every five minutes, as well as before and after the loading dose. Oxygen desaturation (hypoxemia) was defined as a SpO_2_ level below 92% for more than 10 seconds. Management included a chin lift and jaw thrust to ensure a patent airway, and nasal oxygen was increased to 10 L/min. If O_2_ saturation did not improve, the procedure was discontinued, and bag-mask ventilation was provided for 3 minutes. If there was still no improvement, endotracheal intubation was performed, and the patient was excluded from the study.

Bradycardia was defined as a heart rate (HR) of less than 50 beats per minute, while tachycardia was defined as an HR greater than 110 beats per minute. Hypotension was defined as a mean arterial pressure (MAP) less than 60 mm Hg or a 20% decrease from baseline. Hypertension was defined as an MAP greater than 100 mm Hg or a 20% increase from baseline. Bradycardia was managed with IV atropine (0.5 mg). After excluding inadequate sedation, tachycardia and hypertension were treated with a bolus of IV fentanyl (1 µg/kg). Hypotension was managed with a 200 mL bolus of Ringer's solution and Ephedrine (10 mg per dose). Intra-procedure pain was assessed using the Facial Pain Rating Scale (FPS; 0 - 10).

Sedation failure was defined as the need to interrupt the ERCP more than 3 times due to inadequate sedation or hypoxemia. In cases of sedation failure, general anesthesia was administered to complete the procedure. Endoscopist satisfaction was evaluated after the procedure. Inadequate sedation, characterized by patient movement, coughing, or surgeon dissatisfaction, was managed with a bolus of propofol (1 mg/kg over 30 seconds) or dexmedetomidine (0.5 µg/kg over 3 minutes), depending on group allocation, and the maintenance rate was adjusted accordingly. Paracetamol (1 gm) was administered intraoperatively to both groups.

### 3.2. Post-procedure Care

The total doses of propofol and dexmedetomidine, the duration of the procedure, and the interval between the end of the procedure (i.e., removal of the scope) and reaching a BIS score > 90, indicating recovery sufficient for transfer to the post-anesthetic care unit (PACU), were recorded. The Ramsay Sedation Scale (RSS) was used to assess the degree of sedation (11). The six categories are described as follows: 

(1) Awake; either restless or disturbed, or both.

(2) Awake; helpful, focused, and at ease.

(3) Awake but responds only to orders.

(4) Sleeping; responds rapidly to loud noises or a mild glabellar tap.

(5) Sleeping; responds slowly to loud noises or a mild glabellar tap.

(6) Deeply asleep; does not respond to strong auditory stimuli or glabellar tap.

Patients were evaluated using the Modified Aldrete Score (MAS; 0 - 10), which assesses activity, respiration, blood pressure, consciousness, and color. A visual analogue scale (VAS) (0 - 100) was used to measure post-procedure pain. IV morphine (2 mg) was administered to patients with VAS scores > 30, and IV ondansetron (4 mg) was used to address nausea. Patients were required to stay in the recovery room for a minimum of one hour. Discharge readiness was indicated by an Aldrete score of 9 or above (12), where the patient is fully alert, free of unpleasant symptoms (such as nausea or vertigo), demonstrates stable hemodynamic indicators, and can ambulate independently. Potential adverse effects, such as respiratory depression, allergies, coughing, gagging, nausea, and vomiting, were monitored and reported.

The primary outcome was the incidence of hypoxemic events. Secondary outcomes included intra- and post-procedure changes in mean arterial pressure (MAP) and heart rate (HR), BIS and RSS scores, Facial Pain Scale (FPS), time to achieve target sedation, total doses of sedative agents, time to recovery after the procedure, post-procedure Aldrete scale score, VAS score, and incidence of nausea and vomiting, as well as endoscopist satisfaction.

### 3.3. Sample Size Calculation

According to a previous study (13), dexmedetomidine was associated with a lower prevalence of oxygen desaturation compared to propofol (25.6% vs. 8.6%, respectively). To test the hypothesis that the rate of hypoxemia is different between experimental and control subjects with a power of 0.9, we need 101 subjects in the experimental group and 101 subjects in the control group. The Type I error probability for this test is set at 0.05.

### 3.4. Statistical Analysis

Statistical analysis was performed using SPSS v26 (IBM Inc., Chicago, IL, USA). The normality of data distribution was assessed using the Shapiro-Wilk test. For quantitative parametric variables, the mean and standard deviation (SD) were reported and compared using the unpaired Student's *t*-test. The Mann-Whitney test was used for non-parametric data, which were reported as the median and interquartile range (IQR). Qualitative variables were compared using Fisher's exact test or the chi-square test, and were reported as frequency and percentage (%). A result was considered significant if the two-tailed P-value was < 0.05.

## 4. Results

There was no significant difference between the two groups in terms of demographic data and the duration of the procedure. The total dose of propofol was 390.2 ± 64.8 mg, while the dose of dexmedetomidine was 168.6 ± 37.0 µg. There was no significant intergroup difference in the time required to achieve the target sedation level. Dexmedetomidine was associated with a significantly more rapid recovery compared to propofol (P < 0.001) (Table 1). 

**Table 1.** Comparison of Demographic and Clinical Data, Time Till Target Sedation Achieved, and Time Till Recovery Between Propofol Group and Dexmedetomidine Groups ^a^

Variables	Propofol Group; (n = 101)	Dexmedetomidine Group; (n = 101)	P-Value
**Age (y)**	46.0 ± 12.0	45.2 ± 12.8	0.656
**Gender**			0.480
Female	43 (42.6)	48 (47.5)	
Male	58 (57.4)	53 (52.5)	
**Body Mass Index (kg/m** ^**2**^ **)**	26.6 ± 3.4	27.2 ± 3.1	0.139
**ASA Class**			0.471
II	37 (36.6)	42 (41.6)	
III	64 (63.4)	59 (58.4)	
**Duration of procedure (min)**	44 ± 2	45 ± 1	0.186
**Time till target sedation achieved (sec)**	227 ± 95	235 ± 95	0.515
**Time till recovery (min)**	7.0 ± 2.8	5.4 ± 1.8	< 0.001

Abbreviation: ASA, American Society of Anesthesiologists.

^a^ Values are expressed as Mean ± SD or No. (%).

**Figure 1.**
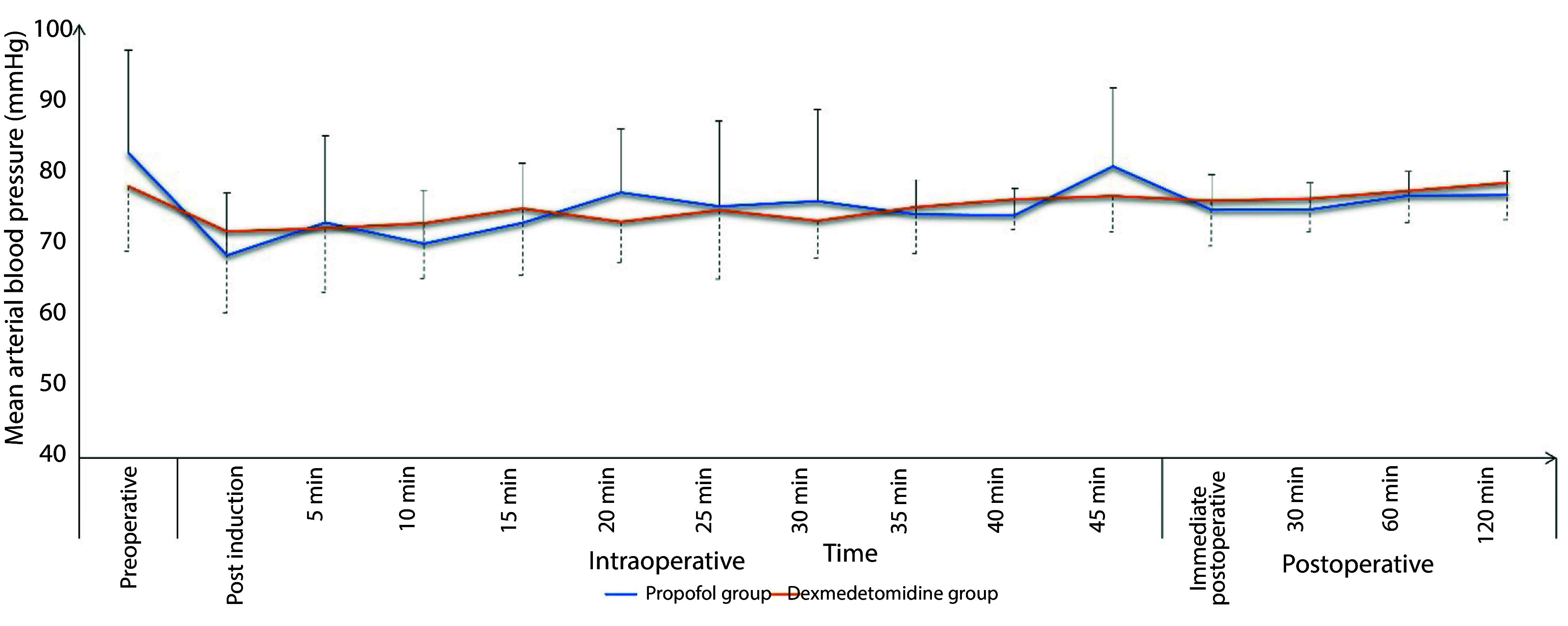
Changes of mean arterial pressure in the propofol and dexmedetomidine groups during and after the procedure (data are presented as mean ± SD).

**Figure 2.**
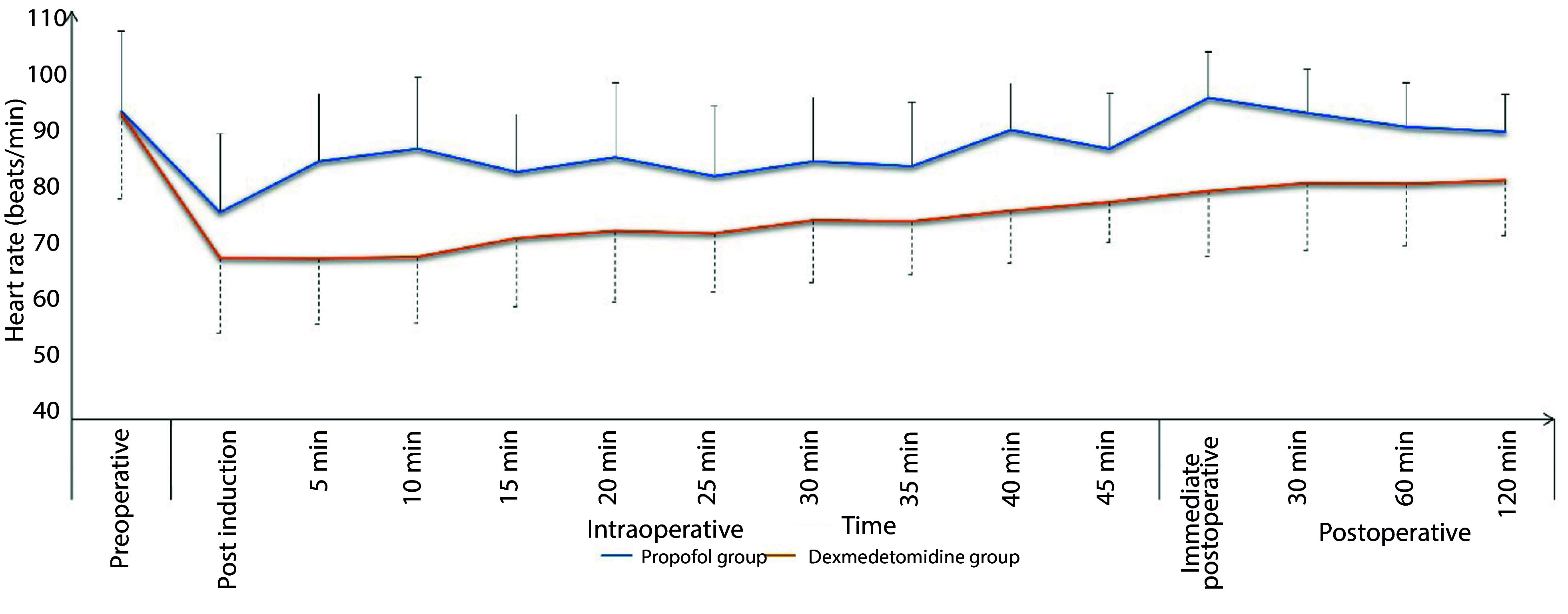
Changes of heart rate in the propofol and dexmedetomidine groups during and after the procedure (data are presented as mean ± SD).

**Figure 3.**
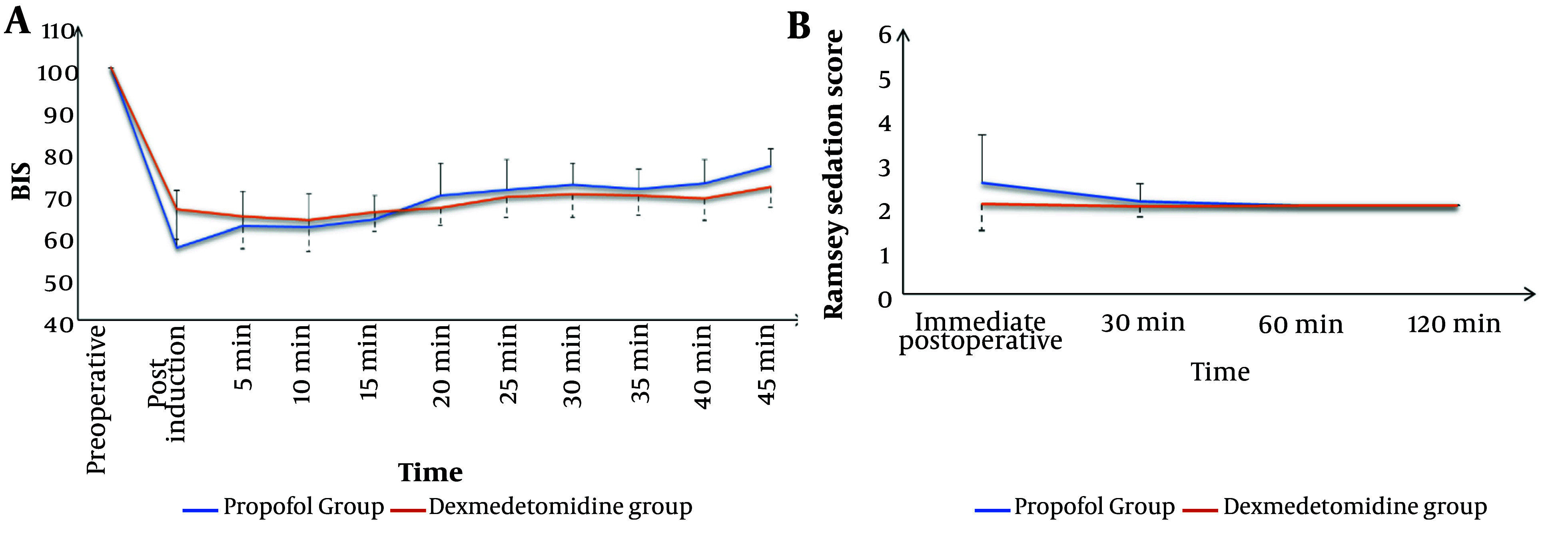
Comparison between propofol group and dexmedetomidine group according to (A), BIS; and (B), postoperative Ramsey Sedation score

The Dexmedetomidine group exhibited a significantly lower incidence of intra-procedural hypoxemic events (14.9% vs. 26.7%, respectively, P = 0.037) and a comparable incidence of hypotension (17.8% vs. 13.9%, respectively, P = 0.441). The total morphine consumption in the first 24 hours was significantly lower in the Dexmedetomidine group compared to the Propofol group (P < 0.001). Sedation failure did not occur in any patient in either group (Table 2). Both groups showed comparable changes in intra- and post-procedure mean arterial pressure (MAP) (Figure 1) and heart rate (HR) (Figure 2). Sedation scores during and after the procedure were also comparable between the two groups (Figure 3). 

**Table 2.** Hypoxemic Events, Morphine Consumption in the First 24 Hours, and Hypotension Events in the Propofol and Dexmedetomidine Groups ^a^

Variables	Propofol Group; (n = 101)	Dexmedetomidine Group; (n = 101)	P-Value
**Number of patients with hypoxemic event**	27 (26.7)	15 (14.9)	0.037
**Number of patients requiring morphine**	93 (92.1)	44 (43.6)	< 0.001
**Total dose of morphine consumption (mg)**	4 (2 - 8)	2 (2 - 6)	< 0.001
**Number of patients with hypotensive event**	14 (13.9)	18 (17.8)	0.441

^a^ Values are expressed as Mean (Range) or No. (%).

**Figure 4.**
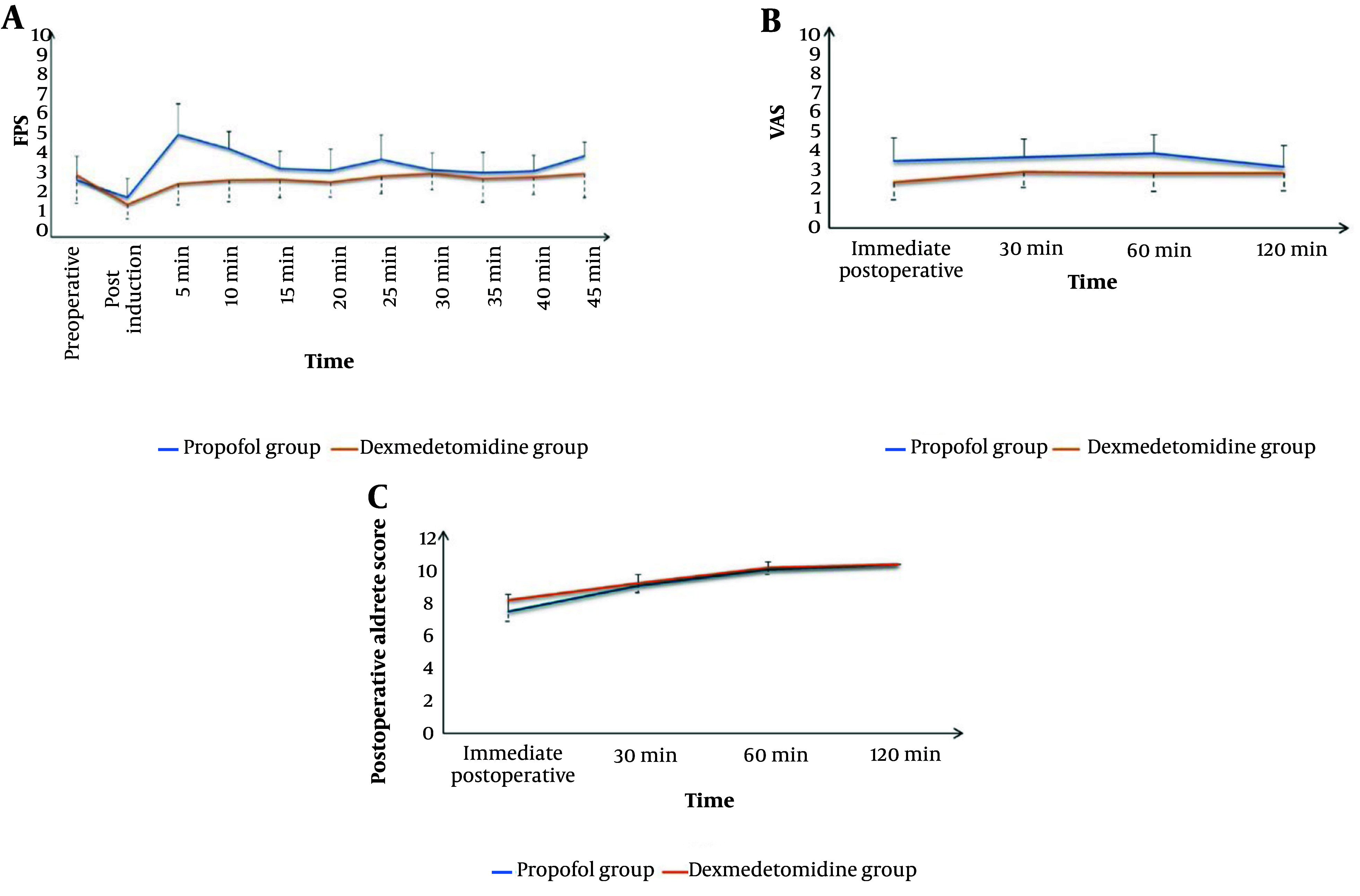
Comparison between propofol group and dexmedetomidine group according to (A), Facial Pain Rating Scale Preop; (B), postoperative visual analogue scale; and (C), postoperative modified Alderete score

**Figure 5.**
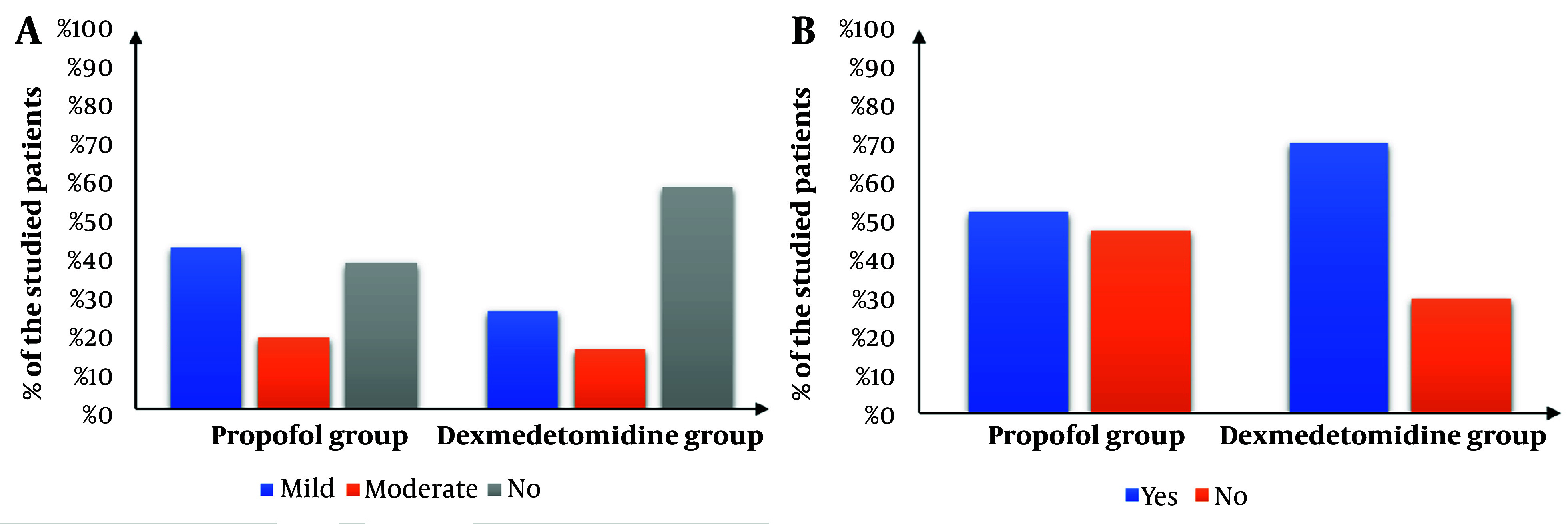
Comparison between propofol group and dexmedetomidine group according to (A), postoperative nausea and vomiting; and (B), endoscopist's satisfaction

The Dexmedetomidine group showed a significantly lower intra-procedure Facial Pain Score (FPS) (P < 0.05) and significantly lower Visual Analog Scale (VAS) scores immediately, 30 minutes, 60 minutes, and 120 minutes after the procedure compared to the Propofol group. Additionally, the Dexmedetomidine group had a significantly higher post-procedure Aldrete score immediately after the procedure (P < 0.001). The scores then became comparable after 30, 60, and 120 minutes (Figure 4). The Dexmedetomidine group also had a significantly lower frequency of postoperative nausea and vomiting (PONV) (P = 0.014) and significantly higher endoscopist satisfaction compared to the Propofol group (Figure 5). 

## 5. Discussion

In this study, deep sedation with dexmedetomidine was associated with a significantly lower incidence of hypoxemic events, better analgesia during (lower FPS) and after the procedure (lower VAS scores), adequate intra-procedure sedation, more rapid recovery, and higher endoscopist satisfaction compared to propofol sedation. Dexmedetomidine had comparable hemodynamic effects, although it was associated with more hypotensive events compared to propofol.

During ERCP, the risk of hypoxemia is known to be higher with deep sedation compared to general anesthesia (14). The incidence of hypoxemia in these cases has been reported to range from 16% to 39% (15). A meta-analysis of propofol sedation in advanced gastrointestinal endoscopy reported a wide variation in hypoxia rates, from 1% to 58%, with a pooled rate of 14.3% (16). Yang et al. (17) reported an incidence of 28% of hypoxia during ERCP with propofol sedation, while more recent studies reported rates as low as 10% (18). Srivastava et al. (19) compared dexmedetomidine with propofol for sedation in ERCP and found hypoxic events in 13% of the propofol group compared to none in the dexmedetomidine group. Similarly, in the current study, dexmedetomidine was associated with a significant reduction in hypoxemic events compared to propofol (14.9% vs. 26.7%, respectively, P = 0.037). Prolonged hypoxia is a common cause of cardiac arrhythmia and coronary ischemia, which can increase the risk of postoperative complications (20).

Therefore, an agent with a lower risk of inducing hypoxemia is needed to address the drawbacks of propofol-induced complications. In the current study, dexmedetomidine is suggested as an alternative to propofol for ERCP in cancer patients due to its sedative and analgesic effects. It induces a sedative response similar to natural sleep, allowing patients to be cooperative when stimulated (21). At an adequate dose, dexmedetomidine provides a level of deep sedation comparable to that of propofol (22).

Two potential advantages of dexmedetomidine were confirmed in the current study: Its analgesic effect (23) and minimal respiratory depression (24). Dexmedetomidine was associated with lower pain scores during and after the procedure compared to propofol. Additionally, the rate of hypoxemia was significantly lower in the dexmedetomidine group. The analgesic properties of dexmedetomidine are mediated through several mechanisms, including spinal, supraspinal, and peripheral actions (25). Its opioid-sparing effect has also been well-documented (26).

Although clinically insignificant, the use of dexmedetomidine was associated with a greater reduction in heart rate in the present study. This can be attributed partly to its vagal mimetic impact and sympatholytic effect due to its action on the α2 adrenoreceptor (27). These findings align with research conducted by Kilic et al. (28) and Inatomi et al. (29), which observed significantly decreased heart rates with dexmedetomidine use.

Hypotensive events were the main adverse outcomes observed with both propofol and dexmedetomidine in the current study. These events occurred in about 14% and 18% of the two groups, respectively, with a non-significant intergroup difference (P = 0.441). Propofol exerts a strong inhibitory effect on the sympathetic nervous system, which is pronounced during the drug's administration and persists throughout the procedure. Similar effects of propofol on blood pressure were reported by Cote et al. (30) and Arian and Ebert (31). However, we noted that the effect on mean arterial pressure (MAP) was relatively more stable in the dexmedetomidine group compared to the propofol group. Accordingly, dexmedetomidine may offer a clinical advantage over propofol in terms of regulating hemodynamic variability. The dosing regimen in the current study was adjusted to mitigate the undesirable side effects of both drugs.

The total amount of injected sedative, and consequently the risk of complications, could be decreased by monitoring the depth of sedation. Various methods, including electroencephalogram (EEG), spectral edge frequency, Bispectral Index, and the Narcotrend device, can be used to track sedation depth. However, EEG alone is not practical during endoscopic procedures due to the time and specialized knowledge required for interpretation. The computer-generated BIS, which ranges from 0 (coma) to 100 (fully awake), provides an indication of sedation depth. A profound sedative state typically requires a BIS of 50 - 60. Paspatis et al. (32) found that using BIS monitoring during ERCP significantly reduced the total amount of propofol administered and shortened the recovery period. Similarly, Al-Sammak et al. (33) demonstrated that employing BIS monitoring reduced the overall sedative dose when using midazolam and meperidine for ERCP.

Throughout the treatment and recovery period, the Facial Pain Score did not show any significant differences. Previous research has shown that dexmedetomidine reduces the need for opioids during surgery and in the post-anesthesia care unit (PACU) (31).

### 5.1. Strengths and Limitations

The primary strength of this study is its large sample size. A unique aspect of the study is the use of deep sedation with dexmedetomidine and propofol in a substantial cohort of cancer patients. A potential limitation is the short follow-up period of 2 hours. Another limitation was the switch from BIS to Ramsay Sedation Scale (RSS) for sedation assessment during the postoperative period, as the BIS monitor was only available in the operating theater. Additionally, the study did not include elderly patients or those with advanced liver or kidney diseases. However, this exclusion was intended to minimize the impact of age and comorbid conditions on the evaluation of the sedative drugs.

### 5.2. Conclusions and Recommendations

Dexmedetomidine can be used as a safe and effective alternative to propofol for the sedation of cancer patients undergoing ERCP. It demonstrates a lower incidence of hypoxic events, better intraoperative sedation, more rapid recovery, and superior analgesic effects, as indicated by lower Facial Pain Scores and postoperative Visual Analog Scale (VAS) scores. It is recommended to use deep sedation during ERCP in cancer patients with either propofol or dexmedetomidine, depending on availability, due to their relative safety and effectiveness. Close intra-procedural monitoring using BIS is advised to control the sedative dose and minimize potential drug complications. We recommend dexmedetomidine for ERCP in cancer patients to leverage its analgesic effects and sleep-like sedation. However, further research is needed to validate these findings and determine the optimal choice of sedative agents for this patient population.

## Data Availability

The dataset presented in the study is available on request from the corresponding author during submission or after publication. The data are not publicly available due to privacy reasons with all included participants.

## Clinical Trial Registration Code:

NCT06409104.
